# Progesterone: A Steroid with Wide Range of Effects in Physiology as Well as Human Medicine

**DOI:** 10.3390/ijms23147989

**Published:** 2022-07-20

**Authors:** Lucie Kolatorova, Jana Vitku, Josef Suchopar, Martin Hill, Antonin Parizek

**Affiliations:** 1 Department of Steroids and Proteofactors, Institute of Endocrinology, Narodni 8, 116 94 Prague, Czech Republic; jvitku@endo.cz (J.V.); mhill@endo.cz (M.H.); 2 DrugAgency, a.s., Klokotska 833/1a, 142 00 Prague, Czech Republic; suchopar@drugagency.cz; 3 Department of Obstetrics and Gynecology, First Faculty of Medicine, Charles University and General Teaching Hospital, Apolinarska 18, 128 51 Prague, Czech Republic; parizek@porodnice.cz

**Keywords:** progesterone, progestin, progestagen, progestogen, neurosteroid, pregnancy, gynecology, miscarriage, preterm birth, menopause, CNS disorder, endocrine disruption

## Abstract

Progesterone is a steroid hormone traditionally linked with female fertility and pregnancy. In current reproductive medicine, progesterone and its analogues play crucial roles. While the discovery of its effects has a long history, over recent decades, various novel actions of this interesting steroid have been documented, of which its neuro- and immunoprotective activities are the most widely discussed. Discoveries of the novel biological activities of progesterone have also driven research and development in the field of progesterone analogues used in human medicine. Progestogen treatment has traditionally and predominately been used in maintaining pregnancy, the prevention of preterm labor, various gynecological pathologies, and in lowering the negative effects of menopause. However, there are also various other medical fields where progesterone and its analogues could find application in the future. The aim of this work is to show the mechanisms of action of progesterone and its metabolites, the physiological and pharmacological actions of progesterone and its synthetic analogues in human medicine, as well as the impacts of its production and use on the environment.

## 1. Introduction

Progesterone (PROG) was one of the first hormones to be identified, and together with estradiol, it is generally known as a female sex steroid [1]. PROG is an endogenous 21-carbon steroid hormone synthesized from cholesterol by way of pregnenolone and is a major gonadal hormone synthetized in the corpus luteum of the ovaries and also by the placenta during pregnancy. To a lesser extent, PROG is also produced at much lower levels by the adrenal cortex, Leydig cells of the testes in men, adipose and other tissues [2,3,4]. As with some other steroids, PROG is also synthesized by the nervous system by neurons and glia (neurosteroid action) and also acts on nervous system tissues (i.e., a neuroactive steroid action). All enzymes necessary for the conversion of cholesterol to pregnenolone and subsequently to PROG are also widely distributed within the brain [5]. PROG can be further metabolized to other neuroactive steroids, of which allopregnanolone is the most important [2].

In this review, we summarize the wide variety of actions of the PROG hormone. Our aim is to show the mechanism of actions of PROG as well as its neuroactive metabolites and the therapeutic potential of PROG and its synthetic analogues not only in gynecology but also in men and the treatment of nervous system disorders.

## 2. Mechanisms of Action

The diverse biological effects of PROG are mediated either via nuclear receptors or non-nuclear receptor mechanisms. The nuclear progesterone receptor (PR) belongs to the steroid subgroup of transcription factors, which activate gene transcription and protein formation. After crossing the cell membrane, PROG binds to the ligand binding domain of nuclear PR and causes a specific conformational change. The receptor-steroid complex dimerizes and, interfering with various other transcription factors, interacts with promoters containing PROG-responsive elements within hormone-regulated target genes [6]. This classic genomic mechanism of steroid action involving mRNA and steroid synthesis is slow, lasting over hours or days, and is the main regulator of female reproduction.

There are two major progesterone receptors, progesterone receptor A (PR-A, with a length of 116 kDa) and progesterone receptor B (PR-B, 4 kDa, lacking 164 amino acids at the N-terminus), which are expressed equally under physiological conditions. Both receptors are coded by a single gene on chromosome 11 and controlled by the proximal PR-A promoter region [7]. PR-B has a unique N-terminal segment that contains a third activation domain. Both forms of PR bind the same steroid hormones with similar binding activities, but they have different transcriptional activities. Thanks to its third activation domain, PR-B is, in general, a much stronger activator than PR-A. It is thus likely that PR-A acts predominately as a repressor and PR-B as an activator. A disrupted balance between PR-A and PR-B may lead to several gynecological pathologies, for example, endometriosis and endometrial hyperplasia [7,8]. It has been shown that PR-A can repress not only the transcriptional activity of PR-B but also transcription mediated by the estrogen receptor and glucocorticoid and mineralocorticoid receptors. In the myometrial tissue, a third progesterone receptor C (PR-C) has also been documented [6,9,10].

**Figure 1:**
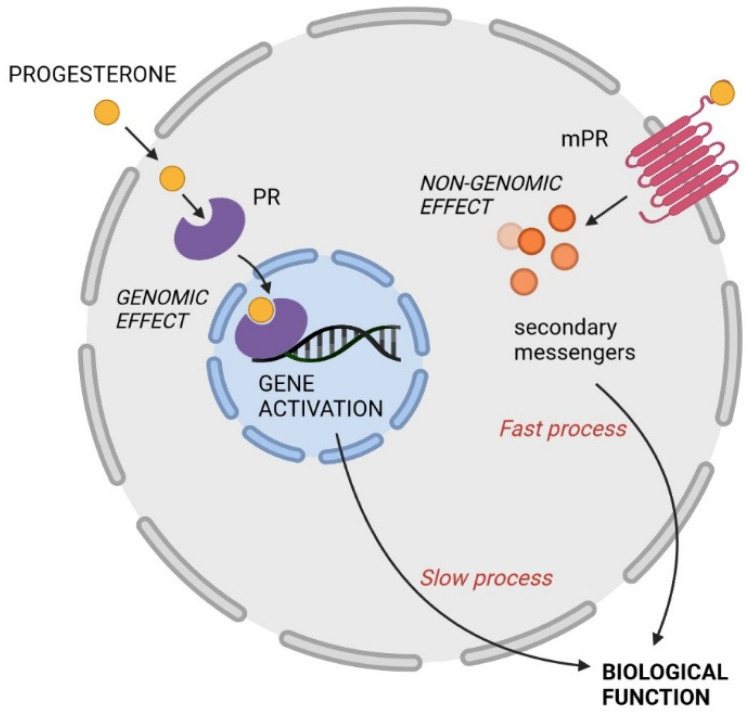
Modes of progesterone action by genomic and non-genomic pathways. mPR—membrane progesterone receptor. Created with BioRender.com.

The non-genomic effects of PROG are generally rapid and have been observed across various tissues in specific cellular compartments through the activation of a wide variety of signal transduction pathways, including ion channels, putative cell surface receptors (action within seconds) and cytoplasmic second messengers (action within minutes) [11,12]. The major transduction pathways used by PROG are extracellular signal-regulated kinase (ERK), cAMP/protein kinase A (PKA), protein kinase G (PKG) signaling, Ca^2+^ influx/protein kinase C (PKC) activation, and the phosphatidylinositol 3-kinase (PI3 K)/Akt pathway [10,11]. The non-genomic effects of PROG are summarized in Table 1. Both genomic and non-genomic PROG actions are illustrated in Figure 1.

## 3. Physiological Actions of Progesterone in Females

Female gonads differ from other organs in the human body in their type of enzymatic apparatus and hormones secreted. The ovaries do not contain 21- and 11β-hydroxylases, and thus, they are not able to produce glucocorticoids and mineralocorticoids. PROG produced in the gonads is carried mostly in the blood to exert its biological function. PROG of adrenal origin is largely converted to glucocorticoids and androgens. In the bloodstream, PROG is highly protein-bound to both cortisol-binding globulin (17%) and albumin (80%), while only a minor proportion of PROG stays unbound (free). PROG has a relatively short half-life in the body of only about 5 minutes. Approximately 50% is metabolized to 5α-dihydroprogesterone in the corpus luteum, 35% is metabolized in the liver to 3β-dihydroprogesterone, and 10% is metabolized to 20α-dihydroprogesterone [48]. These metabolites are then conjugated to sulfates and glucuronides and excreted into the urine. When PROG levels are higher (during the luteal phase, pregnancy or with oral administration of PROG), circulating PROG is also converted by renal 21-hydroxylase to deoxycorticosterone, which may result in unwanted mineralocorticoid-like side effects (water retention, swelling) [10].

In fertile women, sex steroids are synthesized cyclically, and their action determines the menstrual cycle. Steroid synthesis starts in the follicles, which are the functional units of ovaries. Each follicle consists of a single oocyte surrounded by somatic granulosa cells. These are called primordial follicles, and each woman has a finite supply of these follicles starting at birth. Within the first menstrual cycle, a selected amount of primordial follicles start to develop and form so-called pre-antral follicles. During early development, the follicles recruit theca cells, which become vascularized and secure the transport of hormones. Under the stimulation of luteinizing hormone (LH), these theca cells start to synthesize androgens, which are converted to estrogens in somatic granulosa cells via stimulation of follicle-stimulating hormone (FSH) and the aromatase system. The theca cells also synthesize PROG, which acts directly on granulosa cells and promotes follicular growth. When a growing follicle reaches 200–300 μm in diameter, a fluid-filled cavity containing follicular fluid starts to form; these are called antral (secondary) follicles. As the follicle matures, the concentration of estradiol and PROG reaches levels 1–10,000-fold higher than in circulation. In addition, Antimüllerian hormone, inhibin-A and inhibin-B are also synthesized by the granulosa cells. Inhibin-B has been suggested to attenuate pituitary FSH secretion and may augment theca cell androgen production in synergy with LH. These mechanisms are likely to play important functions in the process of selecting the dominant follicle (Graaf follicle) [49]. The dominant follicle also synthesizes PROG and may reach up to 2 cm in diameter. These processes occur in the follicular phase of the menstrual cycle (up to days 12–14). 34–36 hours after the LH surge, ovulation occurs, with the dominant follicle passing into the Fallopian tube where it can be fertilized. The dominant follicle becomes the corpus luteum, which synthesizes PROG in the luteal phase (after ovulation to the end of the cycle) [50]. In the corpus luteum, PROG is synthesized in two enzymatic steps. The first is the conversion of cholesterol to pregnenolone in the mitochondria (cholesterol side chain cleavage enzyme P450scc), and the second is the conversion of pregnenolone to PROG (3β-hydroxysteroid dehydrogenase) [2]. The PROG serum concertation in the follicular phase is lower than 1 ng/mL, but after ovulation, its levels reach 10–35 ng/mL. Stress during the follicular phase may slightly elevate PROG levels (up to 1.6 ng/mL). The corpus luteum secretes PROG continually in the early luteal phase, while in the mid-and late-luteal phase, the PROG secretion reflects the LH pulsatile release. The PROG peak is reached in the mid-luteal phase. In the absence of conception, the corpus luteum decays 9–11 days after ovulation [2,10]. PROG plays a key role in endometrial proliferation. The expression of PROG, as well as estradiol receptors, varies during the menstrual cycle. In the follicular phase of the menstrual cycle, the estrogen receptors predominate, decreasing during ovulation due to the suppressive effects of PROG. In the ovulatory phase, PRs increase exponentially and decrease in the late-ovulatory phase.

In the case of conception, the embryo enters the uterus about 2–3 days after fertilization, and implantation begins 5–6 days later. PROG levels do not increase during this period. Later, the corpus luteum produces PROG until the 8th–9th week of pregnancy. Thereafter, PROG starts to be synthesized by trophoblasts, and after the 12th week of gestation, the placenta is the largest source of PROG. In early pregnancy, levels vary between 10–40 ng/mL and rise to 100–200 ng/mL in late pregnancy [51]. PROG is mainly synthesized from maternal LDL cholesterol by the placenta, with only a minor proportion formed by fetal steroidogenesis. PROG promotes endometrial maturation and uterine vascularization in the pre-implantation phase.

PROG is metabolized primarily in the liver by 5α-reductase and 3β-hydroxysteroid dehydrogenase, resulting in a number of metabolites discussed later. However, the enzymes necessary for PROG metabolism are present in other tissues, especially the corpus luteum, adipose tissue, vaginal mucosa, skin and also in the brain [2,3,52].

## 4. Progesterone and Its Other Effects

### 4.1. Analgetic Effects of Progesterone

Many studies have suggested an important influence of sex differences on pain perception from neonates to adults. This disbalance between men and women is thought to be caused by differences in the response to opioids and steroid hormones. Sex steroids have been shown to affect central opioid activity, while steroid changes in pregnancy can also modulate the opioid system.

Besides the robust expression of PR in the female reproductive tract and mammary glands, PRs are also widely expressed in the nervous system. The study of Kondo et al. demonstrates the importance PRs in the CNS for the pathogenesis of neuropathic pain [53]. It has also been observed that increased levels of PROG can trigger activation of the spinal cord opioid system, increase the release of endogenous opioids and decrease sensitivity to pain [54]. Changes in pain sensitivity as a result of PROG administration are still controversial. Recent results indicate that exogenic administered PROG causes an increase in the expression of delta-opioid receptors in the spinal cord [54]. This is in accordance with a previous study that demonstrated the increased density of delta opioid receptors in the arcuate preoptic area and nucleus after the administration of PROG and estrogen [55].

In 1941, Hans Selye et al. reported that intraperitoneal injections of PROG in rats induced a prompt anesthetic effect, showing for the first time the rapid non-genomic action of PROG at the tissue level [56,57]. In 2006, Kuba et al. [58] performed a study on rats and suggested that the biological basis for sex-specific differences in pain responses resides in the interactions between estradiol and PROG and the activation of pain-related pathways in the CNS. In 2018, Vincent et al. [59] hypothesized that the high PROG state indicative of ovulation would be associated with a reduction in the pain experience. They subsequently examined serum PROG, estradiol, testosterone and cortisol in women at 2 phases of the menstrual cycle: 9 individuals were observed immediately before ovulation (high estradiol and low PROG; days 10–12 of the menstrual cycle), and 8 individuals were observed during the luteal phase (high PROG, high estradiol; days 20–22 of the menstrual cycle). They used commercially available tests confirming ovulation. Probands underwent test monitoring of pain intensity in these two phases of the menstrual cycle, using a thermal resistor to deliver painful thermal stimuli in the arm. They found that pain unpleasantness ratings in response to the pain stimuli that reflect the affective component of pain were significantly lower before ovulation (high PROG) compared to in the luteal phase (low PROG). Higher PROG was associated with a lower affective pain component, reduced activation within the emotion processing network in response to painful stimuli, as well as with decreased connectivity within the emotion regulation network. They described a state of “luteal analgesia”, during which the physiologically high levels of sex steroids seen after ovulation are associated with a specific reduction in the emotional component of pain and reduced brain activation in response to the pain stimuli. Given the availability of exogenous progesterone pharmaceuticals, it may be possible to harness such benefits therapeutically. 

Further clinical studies in humans have also confirmed that sensitivity to pain in women decreases in the luteal phase (high PROG) compared to the follicular phase (low PROG) [58,60,61,62,63]. Decreased pain sensitivity during pregnancy has been demonstrated both in preclinical and clinical studies. Hormonal actions have been implicated as a possible mechanism for pregnancy-induced analgesia. Preclinical studies have shown that supraphysiologic doses of PROG, estradiol and oxytocin can induce antinociception [64]. In 2016, Frölich et al. [64] investigated whether nonlaboring pregnant women have a higher pain threshold and tolerance at term of delivery than 4-8 weeks post-partum. They also studied whether changes in pain perception correlated with changes in hormone levels (PROG, estradiol, oxytocin); nevertheless, they were unable to confirm any of the proposed hypotheses. However, two years earlier, Lee et al. [65] showed that plasma PROG levels were negatively correlated with sevofuran consumption under general Caesarean anesthesia. In another later study, PROG levels also showed a significant negative correlation with a pain index in patients undergoing elective Caesarean section [66], with lower PROG levels and a higher level of pain in patients with higher BMI.

### 4.2. Progesterone as a Neuroactive Steroid

In connection with the discovery of the PROG anesthetic effect, Selye also suggested that 3α-hydroxy metabolites of PROG may be extremely effective sedatives [56]. Although these results were promising, no major progress was made in the following years regarding the mechanism of action of PROG and its use in clinical practice. In the 1970s, Gyermek and Soyka discussed the use of steroids as an anesthetic [67], and Lawrence et al. proposed a hypothesis on the interaction of steroid anesthetics with the lipid layer of neuronal membranes [68]. PROG metabolites, namely 3α, 5α-tetrahydroprogesterone (P3α5α, allopregnanolone), were later considered as barbiturate-like modulators of the γ-aminobutyric acid type A (GABA_A_) receptor [69]. This work initiated intensive research into the effects of neuroactive steroids on GABA_A_ receptors [70,71,72,73,74,75]. Furthermore, effects of neuroactive steroids on glycine [76], N-methyl-d-aspartate (NMDA) [77,78], nicotinic acetylcholine receptors [79] or G-protein coupled receptors [80] have also been found. The term “neurosteroid” (NS) was first used in the Czech literature in 1980 [81] and a year later in France [82] and refers to steroids that affect the nervous system and are also synthesized de novo. They, therefore, occur in the nervous system even in the absence of steroidogenic glands. Neuroactive steroids include all steroids that affect nerve activity, regardless of their site of synthesis [72].

PRs are also expressed in brain areas associated with reproduction and in areas important for cognitive function and emotional processing [83,84]. Animal data suggest that PRs are distributed throughout the hypothalamus, amygdala, hippocampus, thalamus and frontal cortex. The down- and up-regulation caused by PROG only affect those PRs that are inducible by estradiol [2].

#### 4.2.1. Synthesis of Neuroactive Steroids—Progesterone Metabolites

Steroid synthesis begins with the enzymatic conversion of cholesterol to pregnenolone (cytochrome P450scc (CYP11A1)) in mitochondria. Pregnenolone is further converted to steroid hormones in the endoplasmic reticulum, mitochondria and cell cytoplasm [85]. Each steroidogenic tissue contains a specific set of enzymes, thus producing a unique set of steroids and neuroactive steroids. The next step in steroidogenesis includes PROG synthesis. PROG is synthesized through the conversion of pregnenolone by 3β-hydroxysteroid dehydrogenase type 1 and 2. Further reduction is performed by 5α- and 5β- reductases, irreversibly producing 5α/5β-dihydroprogesterone, which is active in the liver, nervous cells and tissues connected with pregnancy and delivery. In the next step, the action of the pluripotent isoenzymes 17β-hydroxysteroid dehydrogenases and aldoketoreductases catalyzing the interconversion of hydroxy-groups to keto-groups and vice versa in positions 3 (3α-hydroxy- ↔ 3-oxo- ↔ 3β-hydroxy), 20 (20α-hydroxy- ↔ 20-oxo-) and 17 (17β-hydroxy ↔ 17-oxo) enables the formation of pregnane metabolites of PROG; these processes are reversible [86].

**Figure 2:**
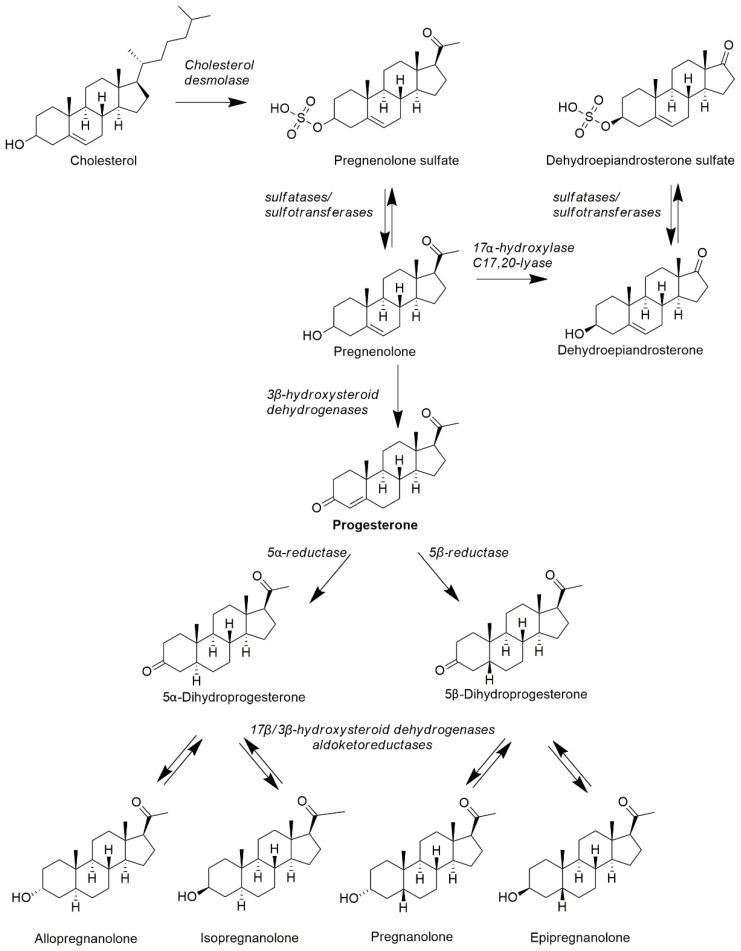
The steroidogenic pathway leading to progesterone and its neuroactive metabolites (pregnane steroids).

By conversion via 5α-reductase, 5α-dihydroprogesterone is synthesized, which is reversibly converted by 17β-hydroxysteroid dehydrogenases and aldoketoreduktases to allopregnanolone (3α-hydroxy-5α pregnan-20-on, P3α5α) and isopregnanolone (3β-hydroxy-5α pregnan-20-on, P3β5α). 5β-dihydroprogesterone is synthesized via 5β-reductase and is then reversibly converted to pregnanolone (3α-hydroxy-5β-pregnan-20-on, P3α5β) and epipregnanolone (3β-hydroxy-5β-pregnan-20-on, P3β5β) by the same pluripotent isoenzymes. These 4 pregnane steroids (allopregnanolone, isopregnanolone, pregnanolone and epipregnanolone) are all neuroactive steroids, of which pregnenolone sulphate and dehydroepiandrosterone-sulphate (DHEAS) are the most abundant in the human circulation. The structures of mentioned steroids and biosynthetic pathways are presented in Figure 2. Pregnane steroids are most abundant in pregnant women as a result of synthesis in the fetoplacental unit [87,88,89]. In non-pregnant women, they are mainly synthesized in the corpus luteum, and the dominant pregnane metabolite is allopregnanolone [90]. Outside the luteal phase and pregnancy, pregnane steroids are also synthesized in the adrenal cortex in the zona fasciculata and in smaller amounts in the zona glomerulosa. The proportion of pregnane steroids of adrenal origin is much lower compared to gonadal [91,92]. Similarly to PROG, pregnane steroids also decrease with age and after menopause [87].

Both gonadal and adrenal pregnane steroids can also cross the blood-brain barrier and influence the steroid metabolome in the CNS [91,93]. The polar conjugates of unconjugated PSs also occur in the bloodstream and possess various biological activities. Pregnane steroids have also been suggested to play roles in situations of stress, with allopregnanolone the most thoroughly investigated. During acute stress, the adrenal glands synthesize great amounts of allopregnanolone, and local production in the brain is increased as well [94]. In contrast, during chronic exposure to stress, serum allopregnanolone levels are lower [95]. Allopregnanolone levels also vary during the menstrual cycle, with serum concentrations temporally following those of PROG with an offset of 2–3 days but with less pronounced differences in the menstrual cycle between phases. The allopregnanolone levels increase from 0.3 ng/mL in the follicular phase to 0.6 ng/mL in the luteal phase. A functionally relevant amount of allopregnanolone can also be synthesized directly in the brain, and it is one of the most widely discussed neurosteroids [2,96,97,98]. The plasma levels of PROG and pregnane steroids are summarized in Table 2.

**Figure 3:**
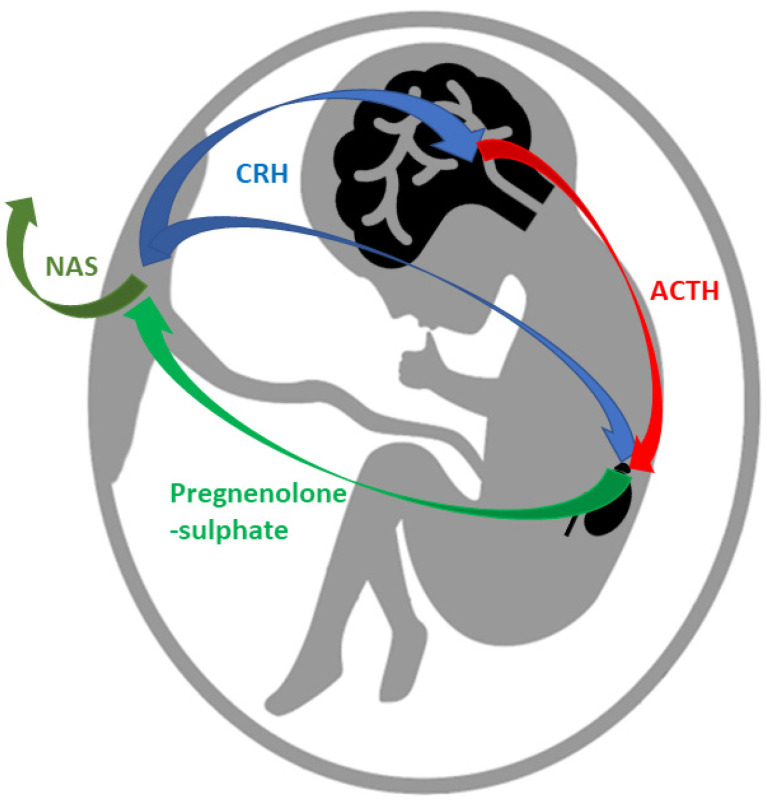
Placenta-fetal interactions in the synthesis of neuroactive steroids. CRH*—*corticotrophin-releasing hormone, ACTH*—*adrenocorticotrophic hormone, NAS*—*neuroactive steroids.

During pregnancy, the concentrations of pregnane steroids rise along with increasing PROG levels. Measurements in the 37th week of pregnancy have shown the following increases: 562-fold higher for PROG; 56-fold higher than the follicular level for isopregnanolone; 37-fold for allopregnanolone, 30-fold for pregnanolone; and 16-fold for epiprenanolone [100]. It has been found that a significant proportion of pregnane steroids present in the maternal circulation comes from steroidogenesis of the fetus. Placental corticotrophin-releasing hormone stimulates their receptors in the hypothalamus, which enables an increase in adrenocorticotrophic hormone and the stimulation of fetal adrenals to synthesize steroid hormones. Fetal adrenals are also stimulated directly from placental corticotrophin-releasing hormone. This binary stimulation (corticotrophin-releasing hormone and adrenocorticotrophic hormone) enables massive levels of steroidogenesis, especially pregnenolone sulfate and DHEAS. These are transported to the placenta, where they are also further metabolized to neuroactive steroids that are secreted to the maternal circulation, resulting in protective, mood-balancing as well as anesthetic effects in the mother’s body. It can be assumed that the fetus produces pregnane steroids for the mother as well [92,101]. A diagram showing the synthesis of pregnane steroids by the fetus is shown in Figure 3.

#### 4.2.2. Mechanism of Action of Pregnane Steroids—Modulation of GABA_A_ and NMDA Receptors

**Figure 4:**
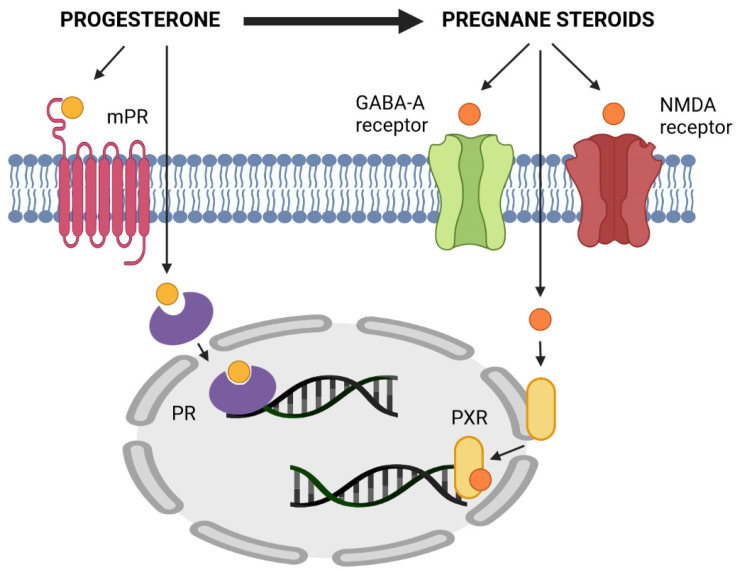
The action on progesterone and its neuroactive metabolites. PR*—*progesterone receptor, mPR*—*membrane progesterone receptor, PXR*—*pregnane X receptor. Created with BioRender.com.

Pregnane steroids mainly act by a non-genomic mechanism, influencing nerve cell excitability by modulating the permeability of ion channels through membrane ionotropic receptors. In the CNS, pregnane steroids bind to and modulate both GABA_A_ (GABA_A_-r) and NMDA (NMDA-r) receptors, with steroid stereoselectivity playing a crucial role in the binding to both receptors. The action of pregnane steroids is illustrated in Figure 4.

For the positive modulation of GABA_A_-r, the presence of a 3α-hydroxy group on the A ring is necessary. The 3α-pregnane steroids (allopregnanolone and isopregnanolone) are positive modulators of GABA_A_-r [70]. These substances act by increasing the frequency and opening time of the chloride channels that are associated with GABA_A_-r. The influx of chloride into nerve cells causes a decrease in their activity. In general, they are thus neuroinhibitors and exhibit sedative, hypnotic, anesthetic, anxiolytic and anticonvulsant properties. These steroids, together with PROG, may be behind the luteal analgesia effect described by Vincent et al. [59]. The 3β-pregnane steroids (pregnanolone and epipregnanolone) and conjugates of all pregnane steroids [31] act as negative GABA_A_-r modulators and thus activate neurons. At least nanomolar concentrations of steroids are necessary for the positive modulation of GABA_A_-r, while antagonists act only in micromolar amounts. Positive modulators of NMDA-r increase the influx of calcium ions into the cell and thus cause neuroactivation, while negative modulators act oppositely. Positive modulators of NMDA-r are polar conjugates of 5α-pregnane isomers (sulphates) [102], while negative modulators of NMDA-r are polar conjugates of 3β-pregnane isomers [103].

It is very likely that there are two basic types of systems affecting the resulting neuromodulatory effect of pregnane steroids. The first is the reversible oxidoreductive equilibrium between 5α/β-dihydroprogesterone and pregnane steroids. The second is the steroid sulfatase and sulfotransferase system when sulfates of 3α5α/β-pregnane steroids and 3β5α/β-pregnane steroids cause neuroactivation, and unconjugated 3α5α/β-pregnane steroids cause neuroinhibition. Moreover, the levels of circulating conjugated pregnane steroids are much higher in comparison with unconjugated pregnane steroids. The sulphates of dehydroepiandrosterone (DHEAS) and pregnenolone (PregS) are also considered neuroactive steroids and cause neuroactivation. GABA_A_ receptors are widely distributed throughout the human central nervous system and can be found within the amygdala, hippocampus and hypothalamus. GABA neurotransmission is the most widespread inhibitory system in the brain. Positive modulation of the GABA_A_-r results in sedative, anxiolytic, anti-convulsant, and neuroprotective properties [2,104]. The effects of pregnane steroids are summarized in Table 3.

As with DHEAS and deoxycorticosterone, PROG and its derivatives are sulfated by SULT2A1 [105]. PROG itself is also an important regulator of SULT1E1 activity, which is responsible for estrogen sulfation [106]. In general, sulfotransferases are regulated by the activation or inhibition of various nuclear receptors and are characterized by significant genetic polymorphism [107].

Changes in PROG levels, which are also reflected in changes in pregnane steroid levels, are likely to cause premenstrual symptoms with an abstinence effect. The abstinence effect caused by a sudden decrease in positive GABA_A_-r modulators occurs rapidly, as does the induction of abuse of these substances. This is because the changes in concentrations of these steroids are accompanied by changes in the expression of the GABA_A_-r subunits responsible for their affinity for these substances. All these processes require precise synchronization, the disruption of which can lead not only to premenstrual syndrome but also to other neuropsychological consequences in physiological and pathophysiological situations such as pregnancy, childbirth, menopause, stress, the application of pharmaceuticals, diseases and many others [108].

#### 4.2.3. Modulation of Other Neurotransmitter Systems

In a comprehensive overview of the effects of PROG in the human body, it is worth mentioning that PROG and allopregnanolone also have the ability to modulate further neurotransmitter systems, such as the serotonergic, cholinergic and dopaminergic systems [2,109].

The serotoninergic system serves various roles, mostly in mood balancing. Sexual behavior and stress responses are also connected with the action of serotonin in addition to the action of steroid hormones [109]. Serotonin is a biogenic amine acting as a neurotransmitter both in the central nervous system as well as in the periphery. In the brain, serotonin is produced within axon terminals, where it is released in response to an action potential and then diffuses across the synapse to activate postsynaptic receptors. Serotonin’s action related to the nervous system is able to modulate mood, cognition, reward, learning and memory. Serotonin receptors also occur outside the nervous system and can modulate other physiological processes such as vomiting, vasoconstriction, sleep, thermoregulation, pain, behavior, sex, feeding, motor activity, biological rhythms and many others [109,110,111]. The serotonin receptors are G-protein-coupled or ligand-gated ion channels and have various subtypes depending on the concrete physiologic action [110].

PROG, as well as estradiol, have been suggested to have a marked effect on the overall function of the serotonin neural system. PROG has been suggested to increase the transmission of serotonin, and conversely, chronic PROG treatment seems to decrease the expression of serotonin receptors in rats [109,112]. Animal studies have confirmed interactions between PROG and estradiol and the serotonergic system [113]. The up- and down-regulation of enzymes affecting the whole monoaminergic neurotransmitter system involving serotonin, noradrenalin and dopamine synthesis have also been associated with PROG. PROG down-regulates monoamine oxidase A mRNA levels in macaque monkeys [114], but up-regulates monoamine oxidase A activity in the hypothalamic areas of rats [115]. PROG and estradiol have been discovered to modify serotogenic responsivity to serotonin-reuptake inhibitors [116]. It was also observed that the co-administration of PROG and cocaine resulted in increased levels of serotonin [117]. Taking into account all these observations, it is clear that there is a connection between PROG levels and serotonin synthesis, and this connection may be behind the mood balance during pregnancy and in the middle of the menstrual cycle.

Dopamine is the key neurotransmitter involved in motor control, learning, motivation, reward, decision-making and working memory. PROG and estradiol can impact dopaminergic neurotransmission via multiple mechanisms. Estradiol and PROG have been observed to affect the number of dopaminergic receptors in rats, with PROG causing inhibition of dopaminergic receptors followed by stimulation in the number of striatal dopaminergic receptors [118]. It has recently been published that 17-hydroxyprogesterone caproate may influence the development of the mesocortical dopamine pathway in a sex- and region-specific manner [119].

### 4.3. Immunomodulatory Effects of Progesterone

A connection between PROG, the main pregnancy steroid, and the immune system has been suspected for more than 70 years. The implantation of the human embryo can be envisioned as an immunological and biological paradox. Immunologically, the embryo is a heterogenous graft, and the uterine immune system and the embryo antigen system (HLA-G) have to collaborate to make the pregnancy possible. Biologically, several different mechanisms must be successively implemented to fuse two different epithelia [120]. Among the many properties of progestogen, the pregnancy-protective role of PROG has also been connected with its immunomodulatory actions. It has been confirmed that lymphocytes of pregnant women express binding sites for PROG [121]. Direct inhibition of K^+^ channels in T cells by PROG might contribute to PROG-induced immunosuppression [27]. There is also a relationship between lymphocyte PR expression and the outcome of pregnancy, with the amount of lymphocyte PRs being significantly lower in women with recurrent abortions and preterm deliveries [122].

The immunological recognition of pregnancy involves the upregulation of the PRs of NK cells in the decidua and in lymphocytes among placental cells. In the presence of PROG in healthy pregnant women, activated lymphocytes and decidual cells synthesize progesterone-induced blocking factor (PIBF). PIBF inhibits arachidonic acid release in lymphoid cells with a subsequent decrease in prostaglandin and leukotriene synthesis. The block of prostaglandin synthesis results in changes in the cytokine balance [121], and PIBF levels in urine have been observed to increase during pregnancy and dramatically decrease following childbirth [123].

Immune cells act via the production and release of cytokines, out of which interleukins form an important subgroup. T-helper (Th) cells are important precursor cells in the cytokine pathway. According to the type of cytokines the immune cells produce, they differentiate into Th1 (pro-inflammatory cytokines, predominately IL-12) and Th2 (anti-inflammatory cytokines, predominately IL-4) lymphocytes, which secrete different interleukins and interferons. Female sex hormones can reinforce this differentiation. In the case of high PROG (and high estrogen) levels, differentiation towards Th2 and the dominance of humoral immunity occurs. There is also a predominance of anti-inflammatory cytokines (IL-4, IL-5, IL-6, IL-10, IL-13, TGF-β, PDGF and LIF) [120]. These Th2 cytokines are necessary for hCG secretion, and they also downregulate Th1 type reactivity. Th1 cytokines are known as pro-inflammatory cytokines and include IL-2, IL-12, IL-18, tumor necrosis factor-α (TNF-α) and interferon-γ (INF-γ) [120]. The block of prostaglandin caused by PIBF results in the reduction of IL-12 production, which has been reported to be elevated in women with pathological pregnancies, and connected with pregnancy termination both in laboratory animals and humans [124].

In the presence of IL-12/IL-4, murine and human T cell differentiation is regulated by the balance of protein kinase C (PKC) and calcium signaling within T cells. Compared to Th1, Th2 shows reduced calcium influx after activation. High PKC activity and low calcium signals favor the development of Th2, while low PKC activity and high calcium signaling favor Th1 development. Phosphorylation of PKC is increased in lymphocytes treated with PIBF, while intracellular levels of calcium are not altered by PIBF. High PKC activity and low intracellular calcium levels favor the development of the Th2 response (IL-4) induced by PIBF [124]. PIBF also affects B-cells, inducing the increased production of non-cytotoxic antibodies and inhibiting the cytotoxicity of NK-cells [120]. PIBF also blocks the NK-cell degranulation and perforin release and inhibits INF-γ, TNF-α and the IL-2 mediated transformation of NK cells into LAK cells (lymphokine-activated killer cells) [120]. By controlling the NK-activity, PIBF exerts an anti-abortive effect.

PROG can also regulate local and systemic inflammation, reducing inflammatory cell infiltration into the cervix and cervical mucus [123]. In vitro studies have also observed the PROG inhibition of human neutrophil degranulation and the generation of free radicals [125,126]. PROG can inhibit mature dendritic cells and the dendritic cell-mediated proliferation of T cells, favoring immature dendritic cells that promote immune tolerance [127]. In addition, PROG and other progestogens also suppress the activity of potent type I interferon-producing dendritic cells [123]. In addition to the tissue-specific effects, PROG has a range of immunosuppressive effects on other innate leukocytes [123].

The human menstrual cycle may naturally serve as a model for the in vivo effects of PROG. The PROG-rich luteal phase is associated with declines in leukocyte proliferation and INF-γ production, as well as a shift toward Th2 cytokine production, with obvious immunoprotective properties. It has also been reported that during respiratory influenza A infections, female mice treated with PROG and levonorgestrel produced fewer antibodies in sera and locally in bronchial-alveolar lavage fluid [128]. It is surprising that while PROG promotes a Th2 dominant immune profile, it also negatively regulates the production of high-affinity antibodies [123].

Overall, PROG mediates a variety of immune adaptations that preferentially promote continued pregnancy. It induces the dominance of Th2 cytokine and anti-inflammatory interleukins and suppresses the pro-inflammatory immune response, occurring both systemically and locally at the maternal-fetal interface and in other biological situations [123].

## 5. Clinical Routes and Applications of Progesterone

As described above, PROG is a key physiological component in the menstrual cycle and in regulating pregnancy. This predetermines its pharmacological use, which needs to be consistent with its physiological effects. PROG provides negative feedback on the secretion of hypothalamic gonadotropin-releasing hormone and pituitary gonadotropins, especially LH. Tissues targeted with PRs are, first of all, the uterus and mammary gland. In the endometrium, PROG plays a key role in the transformation of the proliferative phase into the secretory phase and prepares the endometrium for implantation of a fertilized ovum. PROG also suppresses the contractility of the uterus and its sensitivity to oxytocin and has antiestrogenic and antiandrogenic effects. PROG secretion is regulated by LH, with the secretion of FSH affecting the limbic system through the hypothalamus [129].

Natural PROG is ineffective when administered p.o. because it is highly lipophilic and insoluble in water and is therefore only minimally absorbed. Therefore, local or intramuscular application is commonly used. In 1980, oral micronized PROG in the form of a suspension in oil was introduced. It effectively overcomes the problems with absorption and, at the same time, does not have the adverse metabolic effects of synthetic progestins [130].

PROG is a substrate of liver cytochromes, especially CYP3A4 (and CYP3A5 and CYP3A7 only minorly), which forms 6-hydroxy-PROG and is a minor substrate of CYP2C19, producing 21-hydroxy-PROG [131]. These are important contributors to the high first-pass effect of PROG metabolism in the liver but also in the gut, where 5-8% of the total liver CYP3A4 activity is localized. These cytochrome activities may also explain the lower plasma levels after the p.o. administration of PROG compared to vaginal use. CYP3A4 metabolism should also be considered in the role of drug interactions with PROG administration. For example, ketoconazole is known to reduce PROG metabolism by 95% in vitro. On the other hand, one manufacturer of PROG urges the concomitant administration of CYP3A4 inducers. Unfortunately, there has been a lack of well-designed studies on PROG-drug interactions. In addition, the possible effects of genetic polymorphisms of CYP3A4 and SULT2A1 on PROG metabolism should be considered [132,133,134,135].

There is also confusion concerning the nomenclature of PROG analogues, including natural progesterone, progestagens, gestagens, progestogens and progestins. Progesterone should only refer to the natural hormone produced in the body or the pharmaceutical qualified as body-identical or bioidentical. The terms progestogens, gestagens and progestagens refer to natural or synthetic compounds with progestational activity. The term progestins is used for synthetic compounds able to target PRs; these substances may have different or even opposite pharmacological properties and modes of action [136,137].

### Adverse Side Effects of Gestagens and Progestins

Natural PROG has a unique pharmacodynamic activity and portfolio of side effects compared to synthetic progestins. PROG is a weak agonist of glucocorticoid and androgen receptors and a full antagonist of the mineralocorticoid receptor, which helps to prevent water retention in pregnancy [138,139]. While micronized PROG and especially its metabolite allopregnanolone can modulate GABA_A_ receptors and possess the abovementioned effects in the nervous system, synthetic progestins do not have this potential. Therefore, micronized PROG and synthetic progestins cannot be considered as a single class of medicines [140]. It is worth mentioning that differences in the side effects among natural and synthetic progestins are often misleading in the medical literature, and there is often some confusion concerning the risks of using this medication.

**Figure 5:**
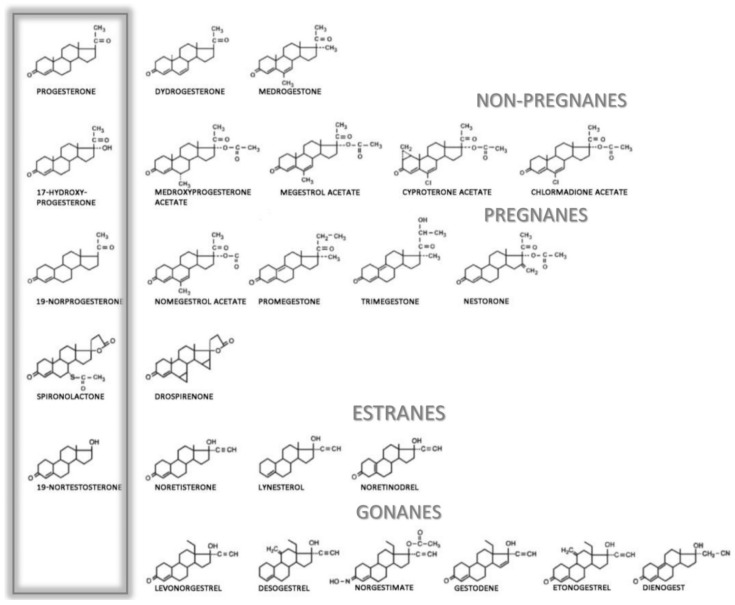
Chemical structures of progestagens used in human medicine.

Depending on their chemical structures, synthetic progestins may bind to PRs as well as to other steroid receptors like glucocorticoid, mineralocorticoid, and androgen receptors, which may cause unwanted side effects. Minor chemical changes in the structure of the progestin molecule can cause important changes in the ability to bind to steroid receptors and may cause various side effects [140]. The binding to steroid receptors is summarized in Table 4, and the structures of progestogens are illustrated in Figure 5.

As with the majority of medicals, undesirable side effects can occur hand-in-hand with the beneficial effects of progestin. The most common side effects are disruption of the menstrual cycle (increased amounts of menstrual bleeding during the regular monthly period, lighter or heavier vaginal bleeding between menstrual periods, or stopping of the menstrual period), headaches, tenderness, nausea and dizziness. Increased depression, fatigue, tiredness, and decreased libido have also been reported. In relation to the androgenic effect of several progestins, acne, oily skin, weight gain, and increased appetite may also be observed. Rarely, a reduction in glucose tolerance with subsequent symptoms and the risk of thromboembolism has been reported [141].

Considering the route of administration is essential in assessments of the pharmacodynamic profile of both natural and synthetic progestins. Oral PROG action is modulated by the presence of gut bacteria and associated enzymes, the intestinal wall and by the liver, whereas these effects are absent in vaginal administration [140]. Oral ingestion of micronized PROG results in rapid absorption and maximal plasma concentrations within 4 h [130]. After ingestion, PROG is metabolized in the gut (5β-reductase activity of gut bacteria) and intestinal wall (5α-reductase activity) and in the liver (5β-reductase, 3- and 20α-hydroxylase activity). The PROG metabolites allopregnanolone and pregnanolone bind the GABA_A_ receptor. This leads to psychopharmacological actions, including the anxiolytic, antidepressant, anesthetic, anticonvulsant and analgesic effects of natural PROG medication [69]. 5α-pregnenedione and 5β-pregnenedione possess anti-mitotic and tocolytic effects. These metabolites probably play a role in negative side effects such as drowsiness, as well as in the therapeutic benefits in some indications such as the alleviation of mood and sleep disturbances and vasomotor symptoms associated with menopause and premenstruum. In contrast, oral PROG has been shown to increase bone formation [142,143,144]. Vaginal PROG administration results in only a small increase in allopregnanolone and no change in 5β-pregnanolone. Normal vaginal bacteria do not possess 5α- and 5β-reductase activity, which is why the CNS is usually only affected when PROG is administered orally. Vaginal administration induces a lower C_max_, and is preferred in the maintenance of pregnancy because of only minor changes in the plasma levels of “psychotropic” metabolites. Vaginal administration also avoids the first-pass effect of the liver [142].

**Table 4:** Patterns of hormonal activities and relative binding affinities to steroid receptors of the main progestogens. Here, 100% binding affinities are related to following steroids: Progesterone receptor (promegestone, 100%), androgen receptor (metribolone R1881, 100%), estrogen receptor (17β-estradiol, 100%), glucocorticoid receptor (dexamethasone, 100%), and mineralocorticoid receptor (aldosterone, 100%). The data are predominately based on animal studies and compiled from the literature [145,146,147,148,149,150,151,152]. The clinical effects of individual substances are dependent on their biological concentrations. The values of relative binding affinities may be inconsistent due to various laboratory protocols, conditions and biological materials used. The anti-estrogen activity of all progestogens is not caused by binding to estrogen receptors but rather by repression of the transcriptional activity of estrogen receptors by PRs.

Progestogen Classification		Progesterone		Androgen			Estrogen			Glucocorticoid		Mineralocorticoid	
		Receptor Binding Affinity	Activity	Receptor Binding Affinity	Androg. Activity	Anti-Androg. Activity	Receptor Binding Affinity	Estrogen Activity	Anti-Estrogen Activity	Receptor Binding Affinity	Activity	Receptor Binding Affinity	Anti-Mineraloc. Activity
**Progesterone derivatives**													
	Natural progesterone	50	+	0	−	+/−	0	−	+	10	+	100	+
	Dydrogesterone	75	+	NA	−	+/−	NA	−	+	NA	NA	NA	+/−
	Medrogestone	NA	+	NA	−	+/−	NA	−	+	NA	NA	NA	−
**17α-hydroxyprogesterone derivatives—Pregnanes**													
	Medroxyprogesterone acetate	115	+	5	+/−	−	0	−	+	29	+	0	−
	Megestrol acetate	65	+	5	+/−	+	0	−	+	30	+	0	−
	Cyproterone acetate	90	+	6	−	++	0	−	+	6	+	8	−
	Chlormadione acetate	67	+	5	−	+	0	−	+	8	+	0	−
**19-norprogesterone derivatives—Non-pregnanes**													
	Nomegestrol acetate	125	+	42	−	+/−	0	−	+	0	−	0	−
	Promegestone	100	+	0	−	−	0	−	+	5	+	0	−
	Trimegestone	330	+	1	−	+/−	0	−	+	9	+/−	120	+/−
	Nestorone	136	+	0	−	−	0	−	+	38 *	−	NA	NA
**Spironolactone derivative**													
	Drospirenone	25	+	2	−	+	0	−	+	0	−	230	+
**19-nortestosterone derivatives—Estranes**													
	Noretisterone	75	+	15	+	−	0	+	+	0	−	0	−
	Lynesterol	NA	+	NA	+	−	NA	+	+	NA	−	NA	−
	Noretinodrel	6	+	0	+/−	−	2	+	+	NA	−	NA	−
**19-nortestosterone derivatives—Gonanes**													
	Levonorgesterel	150	+	45	+	−	0	−	+	1	−	17	+/−
	Desogestrel	1	+	0	−	−	0	−	+	0	−	0	−
	Norgestimate	15	+	0	+	−	0	−	+	1	−	0	−
	Gestodene	90	+	85	+	−	0	−	+	27	+	290	+
	Etonogestrel	150	+	20	+	−	0	−	+	14	+/−	0	−
	Dienogest	5	+	10	−	+	0	−	+	1	−	0	−

(+) effective; (+/−) weakly effective; (−) not effective. NA—data not available. * Nestorone showed significant binding to glucocorticoid receptors; however, it showed no glucocorticoid activity in vivo [153].

## 6. Physiological and Pharmacological Actions of Progesterone and Its Analogues in Selected Physiological and Pathophysiological Conditions

### 6.1. Progesterone in Pregnancy

PROG seems to induce tocolytic and immunosuppressive effects in the areas of contact between the fetal and maternal compartments. Together with hCG and cortisol, it also inhibits the T-lymphocyte-mediated tissue reaction [10,154]. This is called the feto-maternal interface. The maternal immune response has a key role during implantation as well as in the maintenance of early pregnancy, as the maternal immune cells must not attack or reject the fetus during the pregnancy. The anti-inflammatory effects of PROG play an appreciable role. If there is sufficient PROG, pregnancy lymphocytes secrete the so-called progesterone-induced-blocking factor (PIBF), a protein with inhibitory effects on cell-mediated immune reactions. It induces the suppression of T-cell reactions and inhibits NK cells [155]. PROG was shown to have a tocolytic effect in the myometrium. Its effect is concentration-dependent, with only high doses having tocolytic action in early pregnancy. Adequate concentrations in the myometrium are able to counteract the stimulatory activity of prostaglandin and oxytocin [155,156]. During pregnancy, PROG is also believed to act as an immunomodulatory agent via a specific locally produced protein (PIBF) in three ways: (1) by inducing a pregnancy-protective shift from pro-inflammatory Th1 cell-dependent cytokines, (2) by suppressing NK-cell activity in the pregnant uterus, and (3) by increasing the synthesis of asymmetric, anti-abortive antibodies [157].

Indeed, the importance of PROG in pregnancy is reflected in its name, which comes from the Latin pro gestationem. It facilitates blastocyst nesting and is essential in the maintenance of pregnancy [10]. Through progesterone intracellular receptors, PROG stabilizes endometrial activity. It is crucially important during the first trimester, as shown by the fact that the usage of PROG antagonists or dysfunctions of PROG production in the corpus luteum leads to miscarriage in the first trimester [158,159]. During the subsequent two trimesters, the precise functions of PROG are less clear, but levels remain 1–2 orders of magnitude higher throughout the pregnancy compared to levels in non-pregnant women and the drop after delivery of the placenta [160].

In most animal species, the beginning of delivery is associated with a decrease in PROG levels. This decline has not been observed in humans, however. While PROG levels are maintained during labor [161], a “functional” withdrawal of PROG during labor has been noted [51]. PROG probably influences the resting tension of uterine muscles during pregnancy by two possible pathways. The first is related to the diametrically different functions of PR-A and PR-B. While PR-B is an activator of genes that are perceptive to PROG, PR-A is an inhibitor of PR-B expression. At the beginning of labor, there is a significant increase in the expression of the gene for PR-A compared to PR-B, leading to a restriction in PROG effects [162]. Factors responsible for the differing expression of these two PRs are still not clear, but may be prostaglandins or proinflammatory cytokines like TNF-α [163]. Such an increased PR-A expression has also been described in the cervix and amnion [164,165]. The second mechanism of PROG action at the start of labor is connected with its anti-inflammatory effects. At the maternal-fetal interface, PROG acts as an inhibitor of prostaglandin synthesis and pro-inflammatory cytokines (IL-1β and IL-8). It also inhibits the expression of the chemokine MCP-1 (monocyte chemoattractant protein), which is responsible for the influx of monocytes to the myometrium, the further differentiation of monocytes to macrophages, and the production of cytokines and prostaglandins. Increased levels of MCP-1 have been found in the serum of women during labor [160,166]. In addition, decreased PROG levels following delivery trigger milk production [155]. PROG inhibits uterine contractility (inhibiting contractions of the myometrium) and influences remodeling/preparation of the cervix for birth and activation of the amnion. Both oral and vaginal PROG administration are efficacious when a continuation of pregnancy is hampered by immunological factors, luteinic and neuroendocrine deficiencies, or myometrial hypercontractility [155].

### 6.2. Progesterone in Assisted Reproductive Technology

The effects of PROG on the modulation of endometrial structure and function are the basis for successful outcomes in assisted reproductive technology. During stimulated intrauterine insemination, PROG is given after the hCG trigger injection or after the urine LH surge, 400 mg twice a day. If conception is successful, it can be either immediately discontinued or continued until the 12th week of pregnancy. Vaginal administration of PROG has shown increased pregnancy rates compared to women receiving no PROG [167].

Stimulated in vitro fertilization (IVF) cycles are associated with luteal phase deficiency, and supplementation of PROG is therefore necessary. PROG is commenced on the day of oocyte retrieval and can be administered until the 10–12th weeks of pregnancy. The vaginal route of administration is most frequently used in Europe, whereas in the US, i.m. administration is also frequently used. The doses differ depending on the type of cycle used for the embryo transfer. Doses of micronized PROG in natural cycles are 200–400 mg daily, in stimulated cycles (usually stimulation by i.m. FSH and hCG) 400–800 mg daily, and in artificial cycles (women without spontaneous ovarian function, estrogen and progestogen treatment) 600–1000 mg daily [167,168]. Other doses and progesterone forms are reviewed in Labarata et al. [169]. That group also suggests measuring serum PROG levels after embryo transfer. The critical threshold of serum PROG seems to be around 9 ng/mL. Women who exhibited lower serum levels showed a significantly lower ongoing pregnancy rate and higher miscarriage rate. The authors suggest that individual luteal phase treatment on the basis of PROG serum measurement should be of importance [170].

### 6.3. Progesterone in the Prevention of Miscarriage

It has been generally accepted that PROG is effective in cases of insufficient PROG secretion from the first trimester of pregnancy. In 2013, however, Haas and Ramsey reported that there is no evidence to support the routine use of PROG to prevent miscarriage in early and mid-pregnancy, but there seems to be evidence of a benefit of PROG administration in women with a history of recurrent miscarriage [171]. The tocolytic action in early pregnancy was described during high doses of PROG only. Thus, the available data concerning the administration of PROG to prevent miscarriage are not consistent. In 2018, Wahabi et al. published a large review determining the efficacy and safety of PROG in the treatment of threatened miscarriage (a term used to describe continuing abnormal bleeding and abdominal pain during pregnancy). Their study included 7 randomized trials that compared PROG with placebo, no treatment or other treatment in almost 700 women carrying singleton pregnancies from Germany, Italy, Iran, Malaysia, Turkey and Jordan [172]. The analysis suggested that progestogens probably reduce the rate of spontaneous miscarriage.

Concerning the method of administration of progestogens, there is still no clear recommended procedure. Recent data from the USA and China indicate that the oral application of PROG seems to be more effective in the prevention of miscarriage in comparison to vaginal use, but the possible mechanism of efficacy is not discussed [173,174]. Another review exploring data from two large studies from The United Kingdom and Netherlands recommend that pregnant women with vaginal bleeding and a history of 1 or more previous miscarriage(s) be treated with vaginal micronized progesterone 400 mg twice daily until 16 completed weeks of gestation [175]. The same recommendation was actually originally proposed by the same UK group of scientists [176]. A meta-analysis published in 2020 by Chinese authors reviewing the current literature dealing with the benefits of progestogen administration in threatened miscarriage concluded that PROG supplementation may not improve pregnancy outcomes of pregnant women with threatened miscarriage [177].

A study from Singapore published in June 2020 reported the importance of natural levels of PROG in pregnancy. Serum PROG levels were determined in 1087 women between the 5th and 12th weeks of gestation presenting with threatened miscarriage. Women with PROG levels lower than 35 nmol/L were treated with progestogens, while women with PROG levels higher than 35 nmol/L were marked as low-risk and went only to counselling with no progestogen treatment. Both groups were followed up until the 16th week of pregnancy. In the low-risk group, 90.4% had an ongoing pregnancy, but in the high-risk group, 70.8% had a spontaneous miscarriage despite progestogen treatment [178]. A prospective cohort study published in 2018 described the distribution of serum PROG in normal pregnancies and threatened miscarriage in 929 pregnant women. Serum PROG increased linearly with gestational age from 5 to 13 weeks in women with normal pregnancies. Women with threatened miscarriages who experienced spontaneous miscarriages showed a marginal and non-significant increase in serum PROG [179]. In women with threatened miscarriage, serum PROG also increased linearly with gestation, but the median was lower throughout all gestation weeks. The PROG serum levels became normal in ongoing pregnancies after the 13th gestation week because of placental PROG synthesis. Women who miscarried in the first trimester had lower serum PROG (20–30 nmol/L) compared to normal pregnancies [179]. The most recent data from November 2021 included in NICE guideline NG126 found that the vaginal use of 400 mg of micronized PROG in women with threatened miscarriage increases the number of live births [180].

On the basis of these studies, it should be of importance to estimate reference ranges for women who present with threatened miscarriage to predict the risk of spontaneous miscarriage on the basis of PROG levels.

### 6.4. Progesterone in the Prevention of Preterm Birth

Progestogen administration is currently recommended for women at high risk of preterm birth. According to a large study from 2020, vaginally administered PROG (micronized PROG (200 mg daily), 90 mg vaginal gel)) or an intramuscular depot of 17-hydroxyprogesterone caproate (250 mg weekly) is recommended [161]. However, evidence on the influence of PROG on the risks of preterm birth, impact on preterm birth rates, and long-term effects for the baby of implementing these recommendations remains inconclusive.

A Canadian research group concluded in 2019 that vaginal progesterone was the only intervention with consistent effectiveness for preventing preterm birth in singleton at-risk pregnancies overall and in those with a previous preterm birth [181]. Similar conclusions were also obtained in other studies [182,183]. A systematic review published in 2019 [184] included three studies investigating the effect of orally administered PROG and concluded that oral PROG appears to be effective for the prevention of recurrent preterm birth and reductions in perinatal morbidity and mortality rates in asymptomatic singleton gestations with a history of previous spontaneous preterm birth compared with placebo. In 2020, a study by da Fonseca et al. stated that PROG administration halves the risk of preterm birth in singleton pregnancies with a short cervical length. Vaginal PROG administration has also been associated with statistically significant reductions in the risk of respiratory distress syndrome, low birthweight, and fewer admissions to neonatal intensive care units. Vaginal PROG is also recommended in twin pregnancies with short cervical length, in contrast with twin pregnancies with normal cervical length, when PROG vaginal administration may induce spontaneous birth before 32 weeks of gestation [185]. Similar results were published in 2021 by Stewart et al. [186]. These authors also concluded that in contrast to vaginal PROG, there is not enough evidence for using oral PROG and therefore did not support its use.

Finally, i.m. 17-hydroxyprogesterone caproate, a PROG metabolite, was conditionally approved by the Food and Drug Administration (FDA) for the prevention of preterm birth and is currently being investigated [187,188].

### 6.5. Progesterone in Gynecological Pathologies

#### 6.5.1. Luteal Phase Deficiency

Luteal phase deficiency (LPD) is a condition marked by insufficient PROG levels for maintaining a normal secretory endometrium and allowing for normal embryonic implantation and growth. This is one of many etiologies associated with early pregnancy loss and is caused either by impaired function of the corpus luteum or improper development of the dominant follicle destined to become corpus luteum or the aberrant stimulation of a normally developed follicle. Both causes lead to a deficiency of PROG [179]. Chromosomal abnormalities may also be associated with changes in PROG levels [189], and PROG was shown to be lower in pregnancies with trisomy 13 and trisomy 18 [190].

#### 6.5.2. Menorrhagia

Menorrhagia is defined as excessive blood loss during menstruation (more than 80 mL of blood or menses lasting for more than 7 days in several consecutive menstrual cycles). It occurs in reproductive-age women but is more common in perimenopause. The use of PROG and synthetic analogues is well established. Medical management largely consists of using intrauterine progestogen systems (levonorgestrel) or systematic treatment with high doses of oral progestogens.

Progestins are the first-line treatment for heavy menstrual bleeding in an emergency presentation. Oral noretisterone, medroxyprogesterone acetate or micronized PROG (200 mg daily) for 10–20 days in the cycle (depending on the medication) are usually used, with treatment aimed at limiting blood loss. Secondary options include long-term acting progestogene, e.g., an intrauterine levonorgestrel system, etonorgestrel subdermal implant or depot progesterone injections. Treated women have reduced menses, and the medication is also an effective reversible contraceptive. For non-emergency menorrhagia, oral progestogens are preferred; however, the most commonly selected treatment remains combined oral contraceptive pills containing natural estrogen and progestogen. They can effectively regulate the menstrual cycle and reduce excessive bleeding. Specific recommendations for menorrhagia treatments are reviewed in Jewson et al. [191].

Menorrhagia is also treated with non-steroidal anti-inflammatory drugs, antifibrinolytics (tranexamic acid, epsilon-aminocaproic acid) and desmopressin (a synthetic analogue of the antidiuretic hormone vasopressin) [192].

#### 6.5.3. Endometriosis

Endometriosis is defined as the growth of endometrial cells outside the uterus, causing inflammatory disorders associated with chronic pelvic pain and infertility in women of childbearing age. These endometrial cells outside the uterus react to cyclic hormonal changes and can cause the formation of cysts, adhesions and scars. In the normal endometrium, PROG, via PRs, counteracts the action of estradiol and exhibits anti-proliferative and anti-inflammatory properties. As discussed above, there are two PR isoforms, PR-A and PR-B, which are transcribed from a single gene (Pgr) with two alternative promoters. Although the functional interaction between PR-A and PR-B is not required for reproductive activity, experimental studies have indicated that a loss of PR expression or perturbation of PR-mediated signaling is associated with excessive estradiol action in the endometrium and the development of female reproductive diseases, including endometriosis and endometrial hyperplasia [193].

In the normal endometrium, estrogen in the follicular phase acts through the estrogen receptor to increase the transcription of PRs, especially PR-B. PR-B in the luteal phase downregulates estrogen receptors and increases the transcription of 17-β-hydroxysteroid dehydrogenase type 2, which catalyzes the conversion of estradiol to estrone, a less active estrogen. Because of the antagonistic action of PROG to estradiol, progestins are the first-line treatment in endometriosis therapy. In fact, oral progestins such as norethindrone acetate and dienogest have been approved for the treatment of endometriosis and are able to completely eliminate pelvic pain and dysmenorrhea. The usual treatments also combine oral contraceptives; however, there is recent evidence that these actually lead to the progression of this disease [194].

Endometriosis can also be treated by non-steroidal anti-inflammatory drugs, narcotics, gonadotropin-releasing hormone analogues (leuprolide, goserelin, nafarelin) and danazol [195].

#### 6.5.4. Endometrial Hyperplasia

Endometrial hyperplasia is manifested by excessive or abnormal thickening of the uterine lining and is often accompanied by abnormalities in uterine bleeding. It is usually caused by excessive estrogen activity, which may have various reasons (hormonal disbalance, menopause, obesity, etc.) and predominately affects women around menopause. The disbalance between estrogens and PROG may cause the abnormal thickening of the endometrium with alterations of glandular architecture (shape and size). It is classified as a pre-cancerous, non-physiological, non-invasive proliferation of endometrium [196].

PROG exerts its effect on the endometrium through both PRs, which results in the conversion of the endometrium from the proliferative to secretory phase. Progestogens have differing abilities to decidualize endometrium, and this determines their efficacy in reducing or stopping endometrial bleeding. An excessively proliferative endometrium can lead to endometrial hyperplasia, which can progress to or occur together with endometrial carcinoma. Treatment resulting in a reversal from hyperplasia to a normal endometrium is key for the prevention of the development of endometrial cancer [191]. PROG suppresses estrogen-driven growth, and since estrogens are involved in the development of endometrial hyperplasia, progestins mediate their action through PRs. Studies have consistently shown the efficacy of progestin treatment in endometrial hyperplasia. Progestin therapy has an impact on endometrial cells as early as 10 weeks after the initiation of treatment, but most recognize the need for a minimum of 3 months of progestin therapy before assessing for a response. The reversal of endometrial hyperplasia by progestins is thought to occur through activation of PRs, resulting in stromal decidualization and the subsequent thinning of the endometrial lining. The doses and types of progestins for treating endometrial hyperplasia vary individually [197].

Noretisterone, norethynodrel, lynestreol and ethynodiol acetate are very effective in treating endometrial hyperplasia, but they are only rarely used because of their androgenic and metabolic side effects. Medroxyprogesterone acetate is usually used orally or via injection routes. It also has androgenic and glucocorticoidic side effects but is approved by the FDA for its efficiency in endometrial hyperplasia treatment. Chlormadione acetate and nomegestrol acetate are also used, predominately in Europe. Micronized PROG has also been approved by the FDA approved for this medical use. Because of its weaker progestin activity in comparison to synthetic progestins, it is recommended mainly after the initial treatment to prevent the recurrence of endometrial hyperplasia. In this indication, dydrogesterone, chlormadione and medrogestone are also recommended [198].

In postmenopausal women, hysterectomy is also one of the treatment options. Among women hoping for childbirth, treating endometrial hyperplasia is sometimes challenging, and gonadotropin-releasing hormone and its analogues have also been used together with progestins [196].

#### 6.5.5. Secondary Amenorrhea

Secondary amenorrhea is classified as the absence of at least 3 months of menstrual bleeding. It has various causes (functional hypothalamic amenorrhea, polycystic ovary syndrome, hypogonadotropic and hypergonadotropic hypogonadism and others), reviewed in [199,200].

When diagnosing amenorrhea, progestins are used to perform a hormone challenge test, which can help differentiate between anovulation, anatomic and estradiol deficiency as causes. The test shows whether women are able to build up the lining in the uterus. A withdrawal bleed usually occurs two to seven days after progestin application, when the progestin effect disappears [199,201]. If such bleeding occurs, that is taken as evidence that it is a lack of ovulation causing the patient to not have periods. Bleeding will occur only in women with sufficient levels of estrogens.

Options commonly used for this application include oral medroxyprogesterone acetate (10 mg daily), norethindrone (5 mg daily) or micronized progesterone (400 mg daily) used for 7–10 days; a parenteral application of 200 mg of progesterone; or a vaginal micronized progesterone gel (6 days) [199]. In addition, parameters such as thyroid gland function, proper diet, stress, sufficient weight, and excess physical activity (athletes, ballerinas, etc.) are important to check and correct if necessary [202].

#### 6.5.6. Premenstrual Syndrome

In 2012, Ford et al. performed a literature review on the treatment of premenstrual syndrome with PROG. The authors concluded that the various trials were unable to distinguish whether PROG is an effective treatment for premenstrual syndrome or not. No trial was able to identify a subgroup of women who benefited, and none examined claimed successes with high doses [203].

In fact, it was shown that both newer combined contraceptives containing progestins with anti-mineralocorticoid and anti-androgenic activity (drospirenone) and estrogens as well as levonorgestrel intrauterine systems are beneficial for women suffering from premenstrual syndrome. Long-acting gonadotropin-releasing hormone analogues are also highly effective in treating severe premenstrual syndrome. Among non-hormonal treatments, selective serotonin reuptake inhibitors (fluoxetine, sertraline, paroxetine) should be considered along with gonadotropin-releasing hormone analogues as the first line of treatment for severe premenstrual syndrome. Escitalopram, citalopram and venlafaxine have also demonstrated efficacy in reducing premenstrual syndrome symptoms [204].

### 6.6. Progesterone in Menopause

Menopause is defined as a permanent cessation of the menstrual cycle that results from a loss of ovarian activity. In most women (in their 40s and 50s), menopause is preceded by a period lasting several years when cycle irregularities begin, which may mark the onset of the menopausal transition [205]. The postmenopausal phase is characterised by elevated LH and FSH, and low estradiol and PROG. The major circulating estrogen is estrone, which is derived from the conversion of adrenally secreted androstenedione via aromatase situated predominately in adipose tissue. Estrone levels are higher in obese women because of the higher amount of adipose tissue. These women also have a greater risk of estrogen-related malignancies such as breast and endometrial cancer. The elevated gonadotropins may provide an important driver to continued androgen secretion by the ovary, and the administration of estrogens results in the gonadotropin suppression of testosterone secretion. Exogenous estrogens increase the levels of SHBG, so circulating levels of free testosterone may also decrease [205].

The production of estradiol and PROG decreases along with the decline of follicles. Serum estradiol levels fall by 90% and estrone by 70%. Estrogens in postmenopausal women come from the peripheral conversion of androgens produced in ovarian cells and the aromatization of adrenal steroids. Ovarian atrophy occurs later. Given that PROG is mainly produced by the corpus luteum, its levels also decline post-menopause. During the reproductive years, PROG protects the endometrium from excess estrogen stimulation via the direct regulation of estrogen receptors. PROG also exerts a direct intranuclear effect by inhibiting the trophic effect of estrogen on the endometrium. In contrast, estrogen levels post-menopause may remain high enough to stimulate the endometrium, which may lead to a higher risk of endometrial hyperplasia and cancer [206].

Following the decline in female sex hormone levels, there are a number of adverse effects on the body, such as osteoporosis, cardiovascular changes, urogenital conditions, sleep disturbances and many others [207]. For the prevention of these negative menopausal syndromes, hormone replacement therapy is often used. The use of estrogen therapy alone only can be applied to women after hysterectomy. In women with an intact uterus, it is necessary to add progestogen to avoid endometrial hyperplasia that can progress to endometrial carcinoma [208]. Continuous combined hormone replacement therapy involves taking a sustained daily dose of progestogen with estrogen, resulting in the downregulation of endometrial estrogen receptors, resulting in a thin atrophic endometrium. This type of hormone replacement therapy should also be considered for women following hysterectomy for severe endometriosis [209].

The following progestins are commonly used in combined hormone replacement therapy: micronized PROG, dydrogesterone, medrogestone, medroxyprogesterone acetate, megestrol acetate, chlormadione acetate, demegestone, promegestone, trimegestone, nomegestrol acetate, noretisterone acetate, norethindrone, lynesterol, norgestrel, levonorgestrel, norgestimate, and dienogest [137,208].

### 6.7. Progesterone in Men

Defined in the majority of the literature as a female hormone, the importance of PROG in the male endocrine system has remained largely neglected. Testicular and adrenal PROG has been regarded as a physiologically unimportant by-product of steroidogenesis. However, in several conditions, including aging, the serum PROG/androgen ratio increases. The average reference range for serum PROG in healthy men is generally considered under 1 ng/mL, which is similar to in postmenopausal women [210].

The male-specific actions of PROG are predominately membrane-dependent, including a rapid increase in Ca^2+^ resulting in a sperm capacitation/acrosome reaction, LH receptor suppression and the subsequent influence of testosterone biosynthesis in Leydig cells, increased classical PR expressions in the prostate (benign prostatic hyperplasia as well as prostate cancer), interactions with the GABA_A_ receptor complex in the CNS, including sedative and anesthetic actions, and interactions in adipose tissue and the kidneys. PROG-binding membrane proteins have been identified in the liver, sperm and lens epithelial cells [210]. PROG is also one of the steroid hormones that affect spermatogenesis. It has been found that the co-administration of progestins in androgen-based contraceptive pills for men augments the induction of gonadotropin-induced spermatogenic suppression [211]. One study found that the inhibition of sperm production is not caused indirectly by affecting the hypothalamus, but rather the effect of PROG was confirmed directly in the testes [212]. Adding PROG to androgens reduces circulatory concentrations of inhibin and alters the expression of some germ cell-specific genes in human testes [213]. Clinical studies have shown that sperm obtained from oligospermic men had reduced responses to PROG stimulation, suggesting that this membrane effect of PROG can be crucial for the development and fertilizing capacity of sperm [214,215]. It was also reported that PRs may play a role in the regulation of spermatogenesis in humans and that a lack of PR expression in germ cells may be linked to impaired spermatogenesis and could be one possible cause of male infertility [216,217]. PROG is therefore considered to directly act on the testes to regulate spermatogenesis.

### 6.8. Progesterone in the Treatment of CNS Disorders

PROG and progestin hormone therapy for central nervous system disorders is currently an emerging field of regenerative medicine. Multiple sclerosis, amyotrophic lateral sclerosis, spinal cord injury and stroke are diseases that develop via demyelinating, cell death, and/or inflammatory pathological pathways, and progestins have meaningful roles in these processes. The detailed mechanisms are reviewed in Sitruk-Ware et al. (2021) [218], and here we provide only a short overview of the role of PROG in treating CNS disorders.

#### 6.8.1. Multiple Sclerosis

Taking into account the neuroactive and immunoactive properties of PROG, it is not surprising that it has been suggested to play a role in multiple sclerosis. Multiple sclerosis is an inflammatory autoimmune disorder of the CNS causing chronic demyelination and neurodegeneration. PROG was shown to moderately delay the disease onset and reduce the clinical score in a female mouse model [219]. It also attenuated disease severity and reduced the inflammatory response and the occurrence of demyelination in the spinal cord during the acute phase of autoimmune encephalomyelitis [219].

PROG has neuroprotective effects in the CNS, causing an increase in anti-apoptotic mechanisms and cell survival, the regulation of bioenergetic systems, and the induction of neural cell proliferation. PROG also has a regulatory effect on glial cells. It promotes intracellular signaling in the CNS, the proliferation of oligodendrocyte progenitors, and the transcription of key components in the myelin synthesis pathway [220,221]. It has been shown that PROG also influences developmental processes in the nervous system and plays a role in adult neural plasticity [222]. It is involved in the dendritogenesis, synaptogenesis and maturation of cerebellar Purkinje cells, major sites of steroid synthesis in the brain. It has been suggested that PROG and its neuroactive metabolites may play a role in postnatal cerebellar myelination [223]. An important role for PROG in myelination was observed in co-cultures of sensory neurons and Schwann cells [224]. There is also evidence that PROG promotes myelination by oligodendrocytes in the CNS [225,226,227]. PROG also increases the expression of myelin basic protein in mixed glial cell cultures and thus accelerates myelinization [228]. It may also promote the rate of myelin synthesis and indirectly influence myelination via its actions on neurons [229]. Taken together, there is evidence that PROG is an important regulator of myelination during development and myelin repair after pathology in the CNS [230], and that it strongly influences the myelin synthesis in the peripheral and central nervous system [231,232,233]. The exact mechanism by which PROG promotes myelin formation is not yet fully understood; however, the participation of PRs is evident [218,228]. It is also worth noting that there is a shift towards anti-inflammatory cytokine synthesis in the presence of PROG. Pregnancy has a beneficial effect on multiple sclerosis patients by slowing the rate of progression and disability, whereas disease worsening reappears after delivery. It has been proposed that the increased levels of circulating PROG during pregnancy may afford protection against multiple sclerosis [219,234].

The high number of experimental studies documenting neuroprotective and remyelination functions makes PROG seem to be a promising therapeutic option for multiple sclerosis [218,235]. Positive aspects of such treatment include the many active metabolites, long-term experience with their use, and its safety with few negative side effects. One major disadvantage of natural PROG usage is the first-pass liver effect, which degrades a high percentage of PROG ingested. The beneficial effects of PROG outweigh these disadvantages, however, and research into the development of natural progesterone analogues is constantly being driven forward [218].

#### 6.8.2. Amyotrophic Lateral Sclerosis

Amyotrophic lateral sclerosis is a motor neuron disease and, after Alzheimer’s disease and Parkinson’s disease, is the third most common neurodegenerative disorder. PROG has been implicated in various neuroprotective properties, of which longevity [236], muscle strength [237], cell health [238], lowered oxidative stress in the spinal cord, and nitric oxide [236,239] are the most relevant. It has been shown to increase brain-derived neurotrophic factor [237] and normalizes mRNA levels in components of the sodium-potassium pump, which is important for cell nutrition and neurotransmission and is also crucial for mitochondrial health [239]. Moreover, PROG was observed to inhibit the activity of astrocytes, which have predominately deleterious effects in the context of amyotrophic lateral sclerosis because they correspond to increased inflammation. PROG also protects against glutamate excitotoxicity in vitro, one of the major sources of pathology in amyotrophic lateral sclerosis [40]. Synthetic 19-norprogesterone derivatives may also play a role in attenuating motoneuron degeneration and thus have potential in amyotrophic lateral sclerosis treatment [218].

#### 6.8.3. Spinal Cord Injury

Spinal cord injury is defined as damage to the spinal cord due to degeneration, disease or trauma and often causes permanent disability. Currently, there are no therapeutic models with positive effects for spinal cord injury patients, and the only approved pharmacotherapy is the use of pain killers, methylprednisone and glucocorticoids along with physical therapy to alleviate spinal cord injury symptoms [218].

The general neuroprotective and promyelinating effects of PROG described above have been tested in animal models of spinal cord injury. PROG has been shown to reduce harmful effects associated with spinal cord injury, reduce inflammation commonly linked to injury of the CNS, and possibly aid in restoring some function in the spinal cord [240]. As in amyotrophic lateral sclerosis, the anti-inflammatory effects, increase in brain-derived neurotrophic factor, and normalizing of mRNA levels in sodium-potassium pumps are all important in spinal cord injury [218,241]. A clinical trial investigating the administration of PROG and vitamin D documented a significant increase in the recovery of motor function after 6 months [242]. It is clear that therapy with PROG and progestins (especially nestorone) deserves more research and clinical trials.

#### 6.8.4. Stroke

Stroke is the second leading cause of death worldwide. The protective effect of PROG in women before menopause is generally known. In addition to the beneficial effects mentioned previously, PROG has been shown to provide various mechanisms of neuroprotection, including dampening blood-brain-barrier disruptions that may contribute to neuroinflammation [243], reducing cerebral edema in rodent models [244], attenuating the inflammatory response [245], polarizing microglia [246], and ameliorating mitochondrial dysfunction [247]. As in amyotrophic lateral sclerosis, 19-norprogesterone derivatives, including nestrolone, are promising therapeutic agents in this disease [218].

#### 6.8.5. Carpal Tunnel Syndrome

Carpal tunnel syndrome is one of the more common peripheral neuropathies, predominately affecting women after menopause. This syndrome results from the compression of the median nerve of the wrist, and typical symptoms include paresthesia and pain distributed along the median nerve. The disease is often complicated with sensory loss and hand weakness but can be treated with surgery or conservative measures (oral and local steroids, non-steroidal anti-inflammatory drugs, physical therapy and others). Local corticosteroid injection is the principal alternative to surgery, but the efficacy is limited after about one month and has only symptomatic effectiveness [248].

Similarly, as in the CNS, the local synthesis of PROG also plays an important role in myelin formation in the periphery system [231,232,233]. Together with its well-known anti-inflammatory properties, PROG has been suggested to be a therapeutic opportunity in myelin neuropathies like carpal tunnel syndrome. In 2010, Milani et al. initiated a study comparing the effectiveness of intra-carpal injections of either a corticosteroid (triamcinolone acetate) or a single PROG (17α-Hydroxyprogesterone acetate) in patients suffering from carpal tunnel syndrome [248], with complete results published two years later [249]. Both groups exhibited similar reductions in pain scores one month after injections, but while in the PROG treated group, the relief was still present 6 months later, this was not the case in the corticosteroid group. However, another research group later conducted a similar study and found both treatment options to be equally effective [250]. More recently, a group from Sweden concluded that the efficacy of local PROG injections in mild and moderate carpal tunnel syndrome is equal and sometimes superior to corticosteroid injections for relieving symptoms and improving functional and electrophysiologic findings at long-term follow-up [251]. Taken together, these results indicate that intra-carpal injections with long-acting PROG derivatives show promise for long-term relief in carpal tunnel syndrome and are a promising therapeutic option, but further studies are needed.

**Figure 6:**
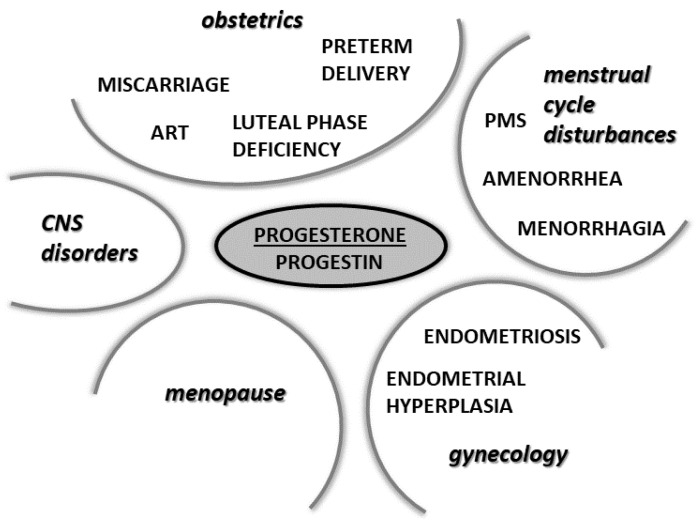
Spheres of influence of progesterone/progestin treatment in women’s health. ART*—*assisted reproductive technology, PMS*—*premenstrual syndrome.

The spheres of influence of PROG/progestin treatment are summarized in Figure 6.

## 7. Endocrine Disruption by Progestins in Wastewater

The endocrine disrupting properties of various chemicals as well as steroids possessing estrogen activity have recently become more widely known and studied. Estrogens and progestins are often used together in medications, and the release of both natural and synthetic progestogens to the environment should also be taken in account [8].

Synthetic progestins are the key ingredient of contraceptives, hormone replacement therapy and other progestogen medication in human as well as veterinary medicine. They are consumed in large quantities and may be excreted in the urine of humans, livestock, and other vertebrates, and introduced into the environment. Recently, synthetic progestins are receiving increasing attention as endocrine-disrupting compounds [252]. In contrast to many other endocrine disrupting chemicals including estrogens, the risks associated with progestogen exposure are not yet fully understood [253,254]. In many cases, progestins should be degraded in wastewater treatment plants, mostly by biodegradation, with a removal efficiency of over 90% [255]. However, inefficient removal of progestins has been reported in central Europe [256]. They have been detected in wastewater and surface waters, livestock manure, soil, and the runoff from farms, and can accumulate in water sediments. They have also been found in paper mill effluents, as steroids can be released during the processing of pine pulp or through the microbial degradation of phytosteroids present in the waste from processing pine trees [257,258,259]. Due to their hydrophobic nature, progestins are able to accumulate in aquatic organisms [260].

Because of these issues, progestins have become a major focus in ecotoxicology over the past decade. The negative effects of progestins as well as many estrogen-active substances especially on male aquatic organisms have been well documented [261,262,263]. Unlike estrogen active endocrine disruptors, however, exposure to synthetic progestins may cause androgenic effects that may disrupt the female hormonal system. In fish, synthetic progestins in picomolar concentration disturb the steroid hormone balance, change the transcription of certain genes, alter sex development and reproduction, and induce female masculinization [264,265,266,267,268]. For instance, exposure to synthetic progestins was shown to result in transcriptional changes in the brain and ovaries of both adult and embryonic zebra fish [253,254]. The accumulation of progestins in children and adults through the consumption of seafood from aquacultures has also been documented [269].

It may be presumed that aquatic organisms are most often exposed to mixtures of multiple contaminants. Much lower concentrations of multiple individual substances can achieve a combined effect that is greater than the action of the substances themselves [270]. This effect has been aptly called “something from nothing” [271]. Therefore, the risks may be higher than indicated by studies dealing only with the effect of a single progestin. For instance, it was found that exposure of common carp to a binary mixture of drospirenone and gestodene caused an increased incidence of intersex individuals (32%) when compared to controls (3%). This was most probably induced by the combined effect of these synthetic progestins, namely their anti-gonadotropic activity and interference with androgen receptors, as well as also potentially with the HPT axis and estrogen receptors [270].

In addition to the current research and undeniably beneficial effects of PROG and its novel synthetical analogues, the potential effects on the environment and other animals should also be taken into account.

## 8. Conclusions

PROG is a steroid hormone acting through multiple pathways in the human organism. In addition to the activity traditionally associated with the female organism and pregnancy, it also plays important roles in male fertility and the neurophysiological condition and mood of both males and females. Treatments using micronized progesterone and its synthetic analogues are of huge importance in gynecology and obstetrics. These substances have allowed a great number of women to conceive a child, prevent premature birth or treat gynecological problems. In addition, the neuroactive effects of PROG and its metabolites hold considerable promise for the treatment of some neurodegenerative diseases, especially those associated with demyelination. Despite the considerable beneficial effects of this hormone, however, its uncontrolled spread in the environment is an important issue to be addressed.

## Figures and Tables

**Table 1:** Non-genomic effects of progesterone across humans and animals. In the given receptor types, mPRs represent membrane progesterone receptors.

Action	Cell/Tissue	Receptor	Signaling Pathway	References
Acrosome reaction/capacitation	Human spermatozoa	mPRs	Ca^2+^, cAMP, G/adenylyl cyclase, mitogen activated protein kinase	[13,14,15]
Steroidogenesis, luteinizing hormone action	Rodent Leydig cells	mPRs	Na^+^	[4,16,17]
Oocyte maturation	Amphibian and fish oocytes	mPRs	G-protein, extracellular signal-regulated kinases, phosphoinositide-3-kinase, cAMP	[18,19,20,21,22,23,24,25]
Immunoregulation	Human T-lymphocytes	mPRs	G-protein, K+ channel	[26,27]
Platelet aggregation	Human platelets	mPRs	Ca^2+^, Src-dependent pathway	[28,29,30]
Anti-apoptotic effect	Rat granulosa cells	mPRs	mitogen-activated protein kinase, Ca^2+^, protein kinase G	[31,32]
Vasoreaction	Rat vascular smooth muscle cells		Ca^2+,^ cAMP	[33]
Actin cytoskeleton remodeling/cell movement	Human umbilical vein endothelial cells	mPRs	G-protein, phosphoinositide 3-kinase, Rho-associated kinase	[34,35]
Muscle contraction	Human intestinal smooth muscle cells	mPRs	Ca^2+^	[36]
Inhibition of proliferation	Smooth muscle cells	mPRs	Src/RhoA—kinases	[37]
Transepithelial resistance	Human fetal membranes	Not determined	not determined	[38]
Activation of transcription factors	Breast cancer	mPRs	extracellular signal regulated kinases, Src/Akt-kinases, phosphoinositide-3-kinases	[34,39]
Neuroprotection	Mouse cerebral cortex, rat hippocampal neurons	mPRs, σ1 receptor	Phosphoinositide 3-kinase, extracellular receptor kinase, Ca^2+^,	[40,41,42,43]
Brain-derived neurotropic factor (BDNP) release	Glia	mPRs	Extracellular signal regulated kinases	[44]
Retinal neuronal activity	Mouse rod bipolar cells	Inositol-triphosphate receptor type 1	Phosphoinositide 3-kinase, Ca^2+^	[45]
Gonadotropin-releasing hormone (GnRH) release	Hypothalamic neurons	mPRs	Not determined	[46]
Lordosis	Ventral tegmental area, mid-brain	GABA_A_/benzodiazepine receptor complexes	Not determined	[47]

**Table 2:** Physiological levels of progesterone and its neuroactive metabolites in fertile women in the follicular and luteal phase of the menstruation cycle and in pregnancy; medians of serum levels are shown. Data were determined using the gas chromatography-tandem mass spectrometry method by Hill et al. [99].

Steroid (nmol/L)	Follicular Phase	Luteal Phase	Pregnancy
Progesterone	1.3	36.2	320
Allopregnanolone	0.51	1.59	32
Isopregnanolone	0.27	0.9	18
Pregnanolone	0.134	0.375	20
Epipregnanolone	0.062	0.168	1.4

**Table 3:** Neuroactive metabolites of progesterone and their action. DHEAS*—*dehydroepiandrosterone sulfate; PregS*—*pregnenolone sulfate.

Receptor	Modulation	Pregnane Isomers	Steroids	Action
GABA_A_	Positive	3α-isomers	Allopregnanolone, pregnanolone	Neuroinhibition
	Negative	3β-isomers	Isopregnanolone, epipregnanolone	Neuroactivation
		Conjugates of all preg. isomers		
			PregS, DHEAS	
NMDA	Positive	Conjugates of 5α-isomers	Allopregnanolone, pregnanolone	Neuroactivation
			PregS, DHEAS	
	Negative	Conjugates of 5β-isomers	Isopregnanolone, epipregnanolone	Neuroinhibition

## Data Availability

Data are available in a publicly accessible repository. The data presented in this study are openly available under reference numbers.

## Abbreviations

PROG: progesterone; PR, progesterone receptor; ERK, extracellular signal-regulated kinase; PKA, protein kinase A; PKG, protein kinase G; PKC, protein kinase C; PI3 K, phosphatidylinositol 3-kinase; PGMRC, PROG membrane receptor component; LH, luteinizing hormone; FSH, follicle-stimulating hormone; CNS, central nervous system; PIBF, progesterone-induced blocking factor; MCP-1, monocyte chemoattractant protein.
